# Saponins from *Camellia sinensis* Seeds Stimulate GIP Secretion in Mice and STC-1 Cells via SGLT1 and TGR5

**DOI:** 10.3390/nu14163413

**Published:** 2022-08-19

**Authors:** Huanqing Zhu, Kaixi Wang, Shuna Chen, Jiaxin Kang, Na Guo, Hongbo Chen, Junsheng Liu, Yuanyuan Wu, Puming He, Youying Tu, Bo Li

**Affiliations:** 1 Department of Tea Science, Zhejiang University, 866 Yuhangtang Road, Hangzhou 310058, China; 2 Department of Tea Science, Zhejiang Shuren University, 8 Shuren Road, Hangzhou 310000, China

**Keywords:** tea seed, Saponin, Theasaponin E_1_, GIP, SGLT1, TGR5

## Abstract

Glucose-dependent insulinotropic polypeptide (GIP) is one of the important incretins and possesses lots of physiological activities such as stimulating insulin secretion and maintaining glucose homeostasis. The pentacyclic triterpenoid saponins are the major active ingredients in tea (*Camellia sinensis*) seeds. This study aimed to investigate the effect of tea seed saponins on the GIP secretion and related mechanisms. Our data showed that the total tea seed saponins (TSS, 65 mg/kg BW) and theasaponin E_1_ (TSE1, 2–4 µM) could increase the GIP mRNA and protein levels in mice and STC-1 cells. Phlorizin, the inhibitor of Sodium/glucose cotransporter 1 (SGLT1), reversed the TSE1-induced increase in Ca^2+^ and GIP mRNA level. In addition, TSE1 upregulated the protein expression of Takeda G protein-coupled receptor 5 (TGR5), and TGR5 siRNA significantly decreased GIP expression in TSE1-treated STC-1 cells. Network pharmacology analysis revealed that six proteins and five signaling pathways were associated with SGLT1, TGR5 and GIP regulated by TSE1. Taken together, tea seed saponins could stimulate GIP expression via SGLT1 and TGR5, and were promising natural active ingredients for improving metabolism and related diseases.

## 1. Introduction

Incretins are a class of hormone peptides released by gut enteroendocrine cells, and they possess lots of physiological functions such as regulating appetite, blood glucose, gastrointestinal motility and lipid metabolism, etc. [1]. Glucose-dependent insulinotropic polypeptide (GIP) and glucagon-like peptide 1 (GLP-1) are two major incretins which respond to nutrients and control glucose homeostasis. Incretin-based pharmacotherapies for diabetes and obesity have received enormous attention. The GLP-1 analogues, GLP-1/GIP dual-agonists and dipeptidyl peptidase-4 (DPP-4) inhibitors that prolong GLP-1 half-life could offer effective treatment for diabetic patients [2,3].

GIP is a 42-amino-acid peptide secreted from K entero-endocrine cells in the duodenum, and its receptor (GIPR) expresses in various organs [4]. GIP can inhibit gastric emptying and motility, control thyroid morphogenesis upon nutrient ingestion, and regulate inflammation and adaptive thermogenesis via restraining myeloid-cell-derived S100 calcium-binding protein heterodimer S100A8/A9 [5,6,7]. In addition, GIP improves energy utilization, reduces inflammation, and exhibits neuroprotective activity in Parkinson’s and Alzheimer ’s disease models [8]. It protects Hippocampal HT-22 cells from glutamate-induced oxidative stress. The underlying molecular mechanisms is that GIP suppresses ferroptosis through activating the mitogen-activated protein kinase (MAPK) pathway [9].

So far, the molecular mechanisms of incretin secretion and related signaling are not very clear. Incretins can be induced via stimulating different receptors by small or macromolecular substances. For example, the sensor receptors for carbohydrates are sodium-dependent glucose transporter 1/3 (SGLT1/3), glucose transporter 2 (GLUT2) and type 1 taste receptor 2/3 (T1R2/T1R3). SGLT1 transfers two Na^+^ molecules when transporting one glucose molecule, causing cell depolarization, opening of voltage-dependent calcium channel (VDCC) and enhancement of intracellular Ca^2+^ concentration, which in turn promotes GLP-1 secretion. Cyclic adenosine monophosphate (cAMP) and Ca^2+^ are considered second messengers for GLP-1 and GIP [10]. Lipid sensor receptors are mainly free fatty acid receptors (FFAR1-4, namely GPR40, GPR43, GPR41 and GPR120) and G protein-coupled receptor 119 (GPR 119). The known sensor receptors of proteins, polypeptides and amino acids are calcium-sensitive receptor (CaSR), T1R1/T1R3G, small peptide transporter (PepT1) and protein-coupled receptor family C group 6 subtype A (GPRC6A) receptor [11]. Cholic acid can stimulate the secretion of GLP-1 and Peptide YY (PYY) via Takeda G protein-coupled receptor 5 (TGR5) and Nuclear farnesoid X receptor (FXR), respectively [12,13]. Lipopolysaccharide induces GLP-1 secretion via Toll-like receptor 4 (TLR4) on intestinal cells, accompanied by an increase in intracellular Ca^2+^ concentration [14].

Tea (*Camellia sinensis* L. (O) Kuntze) is famous worldwide as a popular beverage. In addition to the buds and leaves, tea seeds have received increasing attention due to their abundant active ingredients, including saponins, fatty acids, flavonoid glycosides and polysaccharides. Tea seed saponins are oleanane-type triterpene saponins, and have beenfound to possess various bioactivities, including regulation of gastrointestinal system, weight reduction, anti-microorganism, anti-inflammation, neuroprotection, anti-allergy properties, etc. [15]. Although tea seed saponins have been found to regulate gastric emptying and gastrointestinal transit, their effects on incretins are still not clear. In this study, the effects of total saponins from tea seeds (TSS) and one of the major saponin compound namely theasaponin E_1_ (TSE1) on GIP secretion were evaluated in vitro and in vivo. Moreover, the underlying mechanisms and signaling networks were also investigated.

## 2. Materials and Methods

### 2.1. Chemicals and Regents

TSS and TSE1 (98% purity) were prepared as previously reported [16]. Briefly, hulled tea seeds were ground into powder and extracted with 70% methanol solution at 70 °C. Then, the crude extract was extracted sequentially with petroleum ether, ethyl acetate (EtOAc) and 1-butanol (n-BuOH), and the n-BuOH fraction was eluted with 30–70% ethanol on D101 macroporous resin to give TSS. TSE1 was isolated from TSS by a reversed-phase preparative HPLC system (GE ÄKTA purifier100, Uppsala, Sweden), and identified by UPLC-PDA-MS/MS, ^13^C- and ^1^H-NMR spectroscopy. Phlorizin (purity ≥ 98%) was obtained from Shanghai GE Biological Technology Co., Ltd. (Shanghai, China). Penicillin, streptomycin and Dulbecco’s Modified Eagle’s Medium (DMEM) were provided by Shanghai Guan & Dao Biological Engineering Co., Ltd. (Shanghai, China). Fetal bovine serum (FBS) was purchased from GBICO (Grand Island, NY, USA). The primary antibodies against TGR5 and β-actin and the HRP-linked secondary antibody (anti-mouse IgG) were obtained from Cell Signaling Technology, Inc. (Danvers, MA, USA).

### 2.2. Animal Experiment

Twenty-seven 4-week-old male ICR mice were provided by the Experimental animal center of Zhejiang University. The mice were housed under standard conditions (20–25 °C, 60–70% relative humidity, 12-h light/dark cycle) with free access to water and food. After a 2-week acclimation period, animals were randomly divided into 9 groups (3 mice per cage). Eight groups were administrated with TSS by gavage at a dose of 65 mg/kg BW, and sacrificed after 0.5, 1, 2, 3, 4, 6, 12 and 24 h. The control group received water only, and were sacrificed immediately after gavage. Small intestines were rinsed with 0.9% saline, frozen in liquid nitrogen and stored at −80 °C. All animal experiments were approved by the Experimental Animals Ethics Committee of Zhejiang University (protocol code 12531).

### 2.3. Cell Culture

The mouse intestinal endocrine STC-1 cells were provided by Shanghai Guan & Dao Biological Engineering Co., Ltd. (Shanghai, China). Cells were cultured in DMEM medium supplemented with 10% FBS at 37 °C, and maintained in a thermostatic cell incubator with 5% CO_2_.

### 2.4. Cell Viability Assay

Cell growth was determined using a MTT cell proliferation and cytotoxicity detection kit (KeyGEN BioTECH, Jiangsu, China). Briefly, cells were seeded into 96-well plates, incubated overnight, and treated with TSE1 (0–10 µM) or phlorizin (0–30 µM) for 24 h. Subsequently, 50 μL of MTT solution (1×) was added and incubated for additional 4 h at 37 °C. Then, the supernatant in each well was removed, and 150 uL DMSO was added to dissolve the MTT-formazan crystals. The absorbance at 490 nm was determined with a microplate reader (BioTek, Shanghai, China).

### 2.5. Ca^2+^ Measurement

The intracellular calcium level was determined using a BBcellProbe F03 Assay Kit (BestBio, Shanghai, China). Cells were incubated with calcium-indicating dye for 40 min at 37 °C, and monitored with excitation at 488–495 nm and emission at 516 nm by a microplate reader (BioTek, Shanghai, China).

### 2.6. Quantitative Real-Time PCR (qRT-PCR) Analysis

The total RNA of tissues and cells was extracted using an Eastep^®^ Super Total RNA Extraction Kit (Promega, Wisconsin, WI, USA). Subsequently, the RNA was reversely transcribed to cDNA by a PrimeScript™ RT Reagent Kit with gDNA Eraser (TaKaRa, Kyoto, Japan) according to the operation manual. qRT-PCR was performed on a StepOnePlus™ Real-Time PCR System (Applied Biosystems, Foster City, CA, USA) with a TB Green^®^ Premix Ex Taq™ (Tli RNaseH Plus) Kit (TaKaRa, Kyoto, Japan), and the comparative Ct method (ΔΔCt) was used for analyzing the relative mRNA expression. The primer pairs for GIP and GAPDH were as follows: 5′-GTGGCTTTGAAGACCTGCTC-3′ and 5′-TTGTTGTCGGATCTTGTCCA-3′ (GIP); 5′-GAAGGTGAAGGTCGGAGTC-3′ and 5′-GAAGATGGTGATGGGATTC-3′ (GAPDH).

### 2.7. Enzyme Linked Immunosorbent Assay (ELISA)

The GIP protein concentrations in the small intestine tissues and STC-1 cell culture supernatant were determined by a Mouse GIP (Gastric Inhibitory Polypeptide) ELISA Kit (Elabscience, Wuhan, China) following the operation manual. The plates were read by a microplate reader (BioTek, Shanghai, China) at 450 nm wavelength.

### 2.8. Western Blot Analysis

Cells were lysed with RIPA lysis buffer (Biosharp, Anhui, China) containing a protease inhibitor mixture (Biosharp, Anhui, China). The protein content was analyzed using a BCA Protein Assay Kit (Meilunbio, Dalian, China). Cell lysates were separated by sodium dodecyl sulfate–polyacrylamide gel electrophoresis (SDS-PAGE) and transferred to polyvinylidene difluoride (PVDF) membrane by a Mini-Protean 3 System (BioRad). Afterwards, the membrane was blocked with 5% defatted milk for 1 h, incubated with the primary antibody at 4 °C overnight, and then incubated with the secondary antibody for 2 h at the room temperature. Protein bands were visualized by a LAS-3000 Image Reader (Fujifilm, Tokyo, Japan) with a BeyoECL Plus kit (Beyotime Biotech Inc, Shanghai, China), and analyzed by the ImageJ 1.52v software (NIH, Bethesda, MD, USA).

### 2.9. Transfection with Small Interfering RNA (siRNA)

STC-1 cells were cultured in 6-well plates for 24 h, and transfected with control siRNA or TGR5 siRNA (Santa Cruz Biotechnology, Inc. TX, USA) using the HighGene transfection reagent (Abclonal, Wuhan, China). After a 6 h transfection period, cells were treated with TSE1 for 24 h and used for Western blot analysis.

### 2.10. Protein–Protein Interaction (PPI) Network Construction

The interaction between target proteins was illustrated by the STRING database and GeneMANIA database. The settings in the String system are as follows: Organism, *Homo sapiens*; Network type, full STRING network; Meaning of network edges, evidence; Active interaction sources, all selected; Minimum required interaction score, high confidence (0.700); Max number of interactors to show, select “no more than 10 interactors” in the first shell and “none” in the second shell. In the GeneMANIA system, all default settings were used.

### 2.11. Statistical Analysis

Data from three independent biological replicates were expressed as means ± standard deviation (SD). Mean comparison between two groups and multiple comparisons were assessed by Student’s t-test and one-way analysis of variance (ANOVA) followed with Student–Newman–Keuls (SNK), respectively. *p* < 0.05 and *p* < 0.01 were considered statistically significant and statistically highly significant, respectively. All statistical analyses were performed using SPSS Statistics 26.0 (SPSS Inc., Chicago, IL, USA). Power analysis was performed by PASS 2021 (NCSS, LLC, Kaysville, UT, USA). One-Way Analysis of Variance Assuming Equal Variances (F-Tests) was used to assess the sample size.

## 3. Results

### 3.1. Effect of TSS on GIP Expression in the Small Intestine of Mice

**Figure 1:**
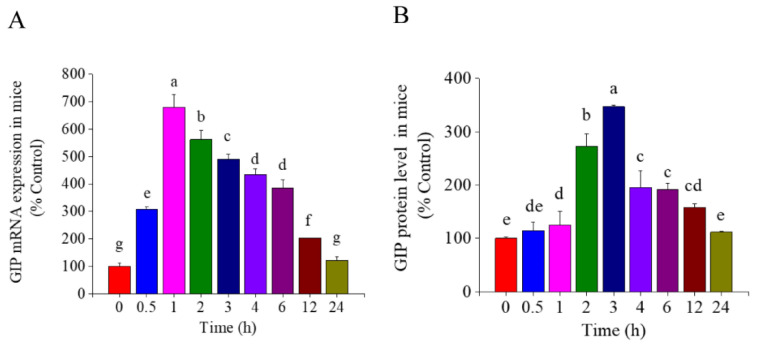
Effect of total tea seed saponins (TSS) on the GIP expression in the small intestine of mice. (**A**) The mRNA expression of GIP determined by RT-PCR. (**B**) The protein level of GIP determined by ELISA. Different letters indicate significant difference among groups (*p* < 0.05).

Mice were administered with TSS by single gavage at the dose of 65mg/kg BW. As shown in Figure 1, the mRNA and protein levels of GIP in the small intestine of mice both significantly increased during the first several hours and then decreased (*p* < 0.05). The highest expression of GIP mRNA and protein occurred at 1 and 3 h, which were 6.8-fold and 2.7-fold higher than that of the control group, respectively. Numerous studies have demonstrated asynchrony in mRNA and protein expression, possibly due to differences in the timing and location of eukaryotic gene transcription and translation.

### 3.2. Effect of TSE1 on GIP Secretion in STC-1 Cells

**Figure 2:**
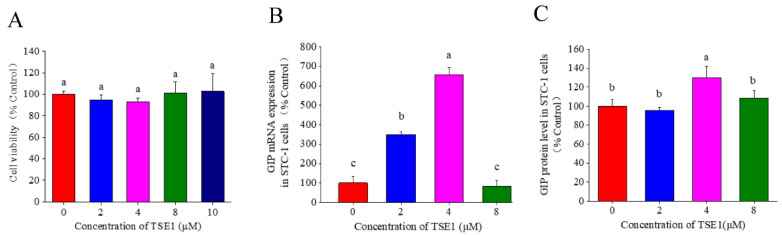
Effect of TSE1 on the GIP expression in STC-1 cells at 24 h. (**A**) Cell viability after 24 h treatment of TSE1 determined by MTT assay. (**B**) The mRNA expression of GIP determined by RT-PCR. (**C**) The protein level of GIP in the cell culture media determined by ELISA. Different letters indicate significant difference among groups (*p* < 0.05).

To further evaluate the effect of tea seed saponins on intestinal GIP secretion, enteroendocrine STC-1 cells were treated with the individual saponin compound TSE1 for 24 h. Figure 2A shows that TSE1 at the concentrations of 0–10 µM did not inhibit the STC-1 cell proliferation (*p* > 0.05), indicating that TSE1was not toxic to cells within this dose range. The mRNA expression of GIP increased to 3.5 and 6.6 times that of the control at 2 and 4 µM of TSE1 (*p* < 0.05), and the protein level in the cell culture media was 1.3-fold higher than control at 4 µM (*p* <0.05) (Figure 2B,C). These data suggest that TSE1 could enhance the GIP expression at relatively low concentrations.

### 3.3. Role of SGLT 1 in TSE1-Induced GIP Expression

**Figure 3:**
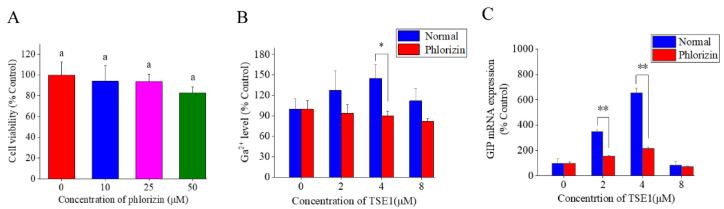
Effect of phlorizin on the Ca^2+^ concentration and GIP expression in STC-1 cells at 24 h. (**A**) Cell viability after 24 h treatment of phlorizin determined by MTT assay. (**B**) The intracellular Ca^2+^ concentration determined by BBcellProbe F03 assay. (**C**) The mRNA expression of GIP determined by RT-PCR. Different letters indicate significant difference among groups (*p* < 0.05). * *p* < 0.05, ** *p* < 0.01, compared with normal group.

Phlorizin, a dihydrochalcone from the bark of pears, apples, cherries and other fruits, is a known inhibitor of SGLT1 [17]. In this work, phlorizin was used to clarify the effect of SGLT1 on TSE1-induced GIP secretion. Figure 3A shows that the cell viability did not change obviously at 10 and 25 µM of phlorizin, and slightly decreased by 17% at 50 µM (*p* > 0.05). Therefore, 25 µM of phlorizin was used in subsequent experiments. As shown in Figure 3B,C, phlorizin could significantly reverse the increase in intracellular Ca^2+^ concentration (*p* < 0.05) and GIP mRNA level (*p* < 0.01) induced by TSE1 at 2 and 4 µM, indicating that SGLT1 played a key role in TSE1-induced GIP expression.

### 3.4. Effect of TSE1 on TGR5 Expression in STC-1 Cells

**Figure 4:**
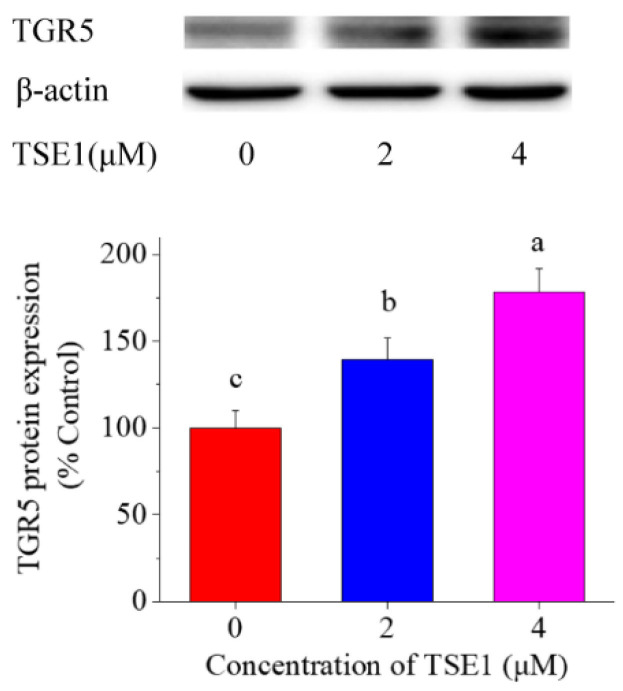
Effect of TSE1 on the TGR5 protein expression in STC-1 cells at 24 h. Protein levels were detected by Western blot, and analyzed by the ImageJ software. Different letters indicate significant difference among groups (*p* < 0.05).

Considering that TGR5 is a target of oleanolic acid, and contributes to the GLP-1 and -2 production [18,19], the effect of TSE1 on TGR5 expression was tested. Figure 4 shows that the protein level of TGR5 was significantly enhanced by TSE1 at 2 and 4 µM in a dose-dependent manner (*p* < 0.05), suggesting that TGR5 might be a target of tea seed saponins.

### 3.5. Role of TGR5 in TSE1-Induced GIP Expression

**Figure 5:**
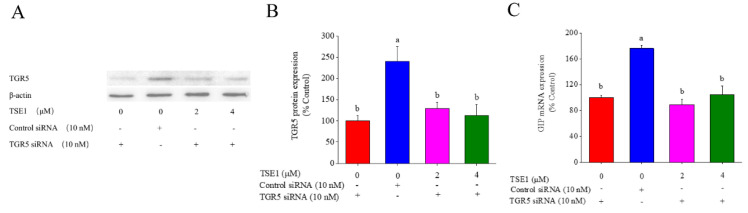
Role of TGR5 in TSE1-induced GIP expression in STC−1cells. (**A**) Effect of TGR5 siRNA on the TGR5 protein expression determined by Western blot. (**B**) Quantification of protein bands analyzed by the ImageJ software. (**C**) Effect of TGR5 siRNA on the GIP mRNA expression determined by RT-PCR. Different letters indicate significant difference among groups (*p* < 0.05).

In order to clarify whether TGR5 is involved in the regulation of GIP by TSE1, TGR5 was knocked down in STC-1 cells by siRNA approach. As shown in Figure 5A,B, TGR5 siRNA effectively reduced the protein expression of TGR5 compared with the controls, meanwhile, it significantly suppressed TSE1-induced upregulation of TGR5 (*p* < 0.05). Figure 5C shows that TSE1 did not enhance GIP mRNA expression after treatment with TGR5 siRNA, indicating that TSE1-induced GIP upregulation through activating TGR5 in the STC-1 cells.

### 3.6. PPI Network and KEGG Analysis

**Figure 6:**
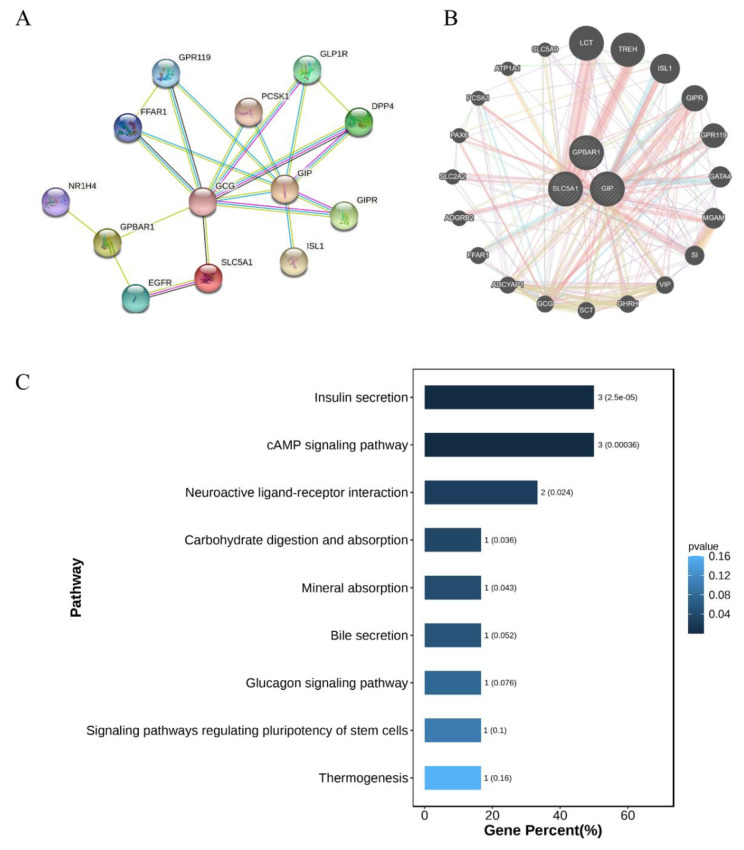
Protein–protein interaction (PPI) network of TGR5 (GPBAR1), SGLT1 (SLC5A1) and GIP. (**A**) The PPI network plotting constructed by the STRING database. (**B**) The PPI network plotting constructed by the GeneMANIA database. (**C**) KEGG enrichment analysis of target proteins.

In order to further explore the regulatory network of TSE1-induced GIP expression, the three proteins, TGR5 (also called G-protein-coupled bile acid receptor 1, GPBAR1), SGLT1 (also called Solute carrier family 5 member 1, SLC5A1) and GIP, were uploaded to the STRING database and GeneMANIA database to construct a PPI network. As shown in Figure 6A,B, six common proteins, including insulin gene enhancer binding protein-1(ISL1), gastric inhibitory polypeptide receptor (GIPR), glucagon (GCG), G protein-coupled receptor 119 (GPR119), free fatty acid receptor 1 (FFAR1) and neuroendocrine convertase 1 (PCSK1), occurred in both databases. Figure 6A shows that GCG was a key node connecting GPBAR1 and SLC5A1 with GIP. Figure 6B shows that GIPR, GPR119 and FFAR1 were related with GPBAR1 and GIP, PCSK1 was associated with SLC5A1 and GIP, and GCG was connected with GPBAR1, SLC5A1 and GIP. These data provided a possible network of protein interactions that involved in the GIP regulation by TSE1. KEGG analysis demonstrated that the six common proteins genes together with GPBAR1 and SLC5A1 were significantly enriched in five pathways including insulin secretion, cAMP signaling pathway, neuroactive ligand–receptor interaction, carbohydrate digestion and absorption, and mineral absorption (*p* < 0.05) (Figure 6C, Table S1), indicating that these signaling pathways are involved in TSE1-mediated upregulation of GIP expression and related biological functions.

## 4. Discussion

The intestine is not only the main site of digestion, but also the largest endocrine organ of human body. The incretin hormones have received increasing attention over the past few decades due to their modulatory effects on appetite, blood sugar, insulin secretion, lipid metabolism, gastrointestinal motility and immune function [20]. These bioactivities confer incretins as the potential therapeutic targets for the treatment of obesity, diabetes and cardiovascular disease [3,21].

Numerous studies have focused on GLP-1 and GIP receptor agonists and DPP-4 inhibitors for development of incretin-based antidiabetic drugs [22]. Recently, some nutrients and natural products such as L-tryptophan, lauric acid, α-linolenic acid and nobiletin were found to regulate glycaemia, gastric emptying, food intake and circadian rhythm via incretin stimulation [23,24,25]. Two saponins, ginsenosides and glycyrrhizic acid, have been reported to increase GLP-1 secretion, which may be related to their antidiabetic effects [13,26]. The triterpene saponins from tea seeds could modulate gastrointestinal system and blood lipids in vivo. However, little is known about their effects on incretin. In this work, it was demonstrated that TSS (65 mg/kg BW) and a major saponin compound, TSE1 (2 and 4 µM), could significantly increase the mRNA and protein levels of GIP in the mouse small intestine and STC-1 cells within 24 h (Figure 1 and Figure 2). These results suggest that stimulation of GIP secretion might be an important mechanism by which tea seed saponins regulate intestinal function and metabolism.

SGLT1 (SLC5A1) is a key glucose transporter regulating glucose absorption in the gastrointestinal tract, and is considered a potential target for treating obesity and diabetes [27]. Several researches have demonstrated that SGLT1 participates in glucose- and high-fat-dependent GIP secretion in the normal, obese and diabetic states [28,29,30]. Activation of SGLT1 at the brush border of the small intestine leads to Na^+^ influx, membrane depolarization, and opening of Ca^2+^ voltage-gated channels, resulting in intracellular Ca^2+^ accumulation and secretion of intestinal peptides by enteroendocrine cells [31]. SGLT1 inhibitors block glucose-mediated GLP-1 secretion, and reduce intracellular cAMP and Ca^2+^ concentrations [32]. Our data showed that the SGLT1 inhibitor phlorizin (25 µM) could significantly reverse the TSE1-induced enhancement of intracellular Ca^2+^ concentration and GIP mRNA expression (Figure 3), suggesting that TSE1 regulates GIP expression at least in part by triggering SGLT1 in the small intestine.

TGR5 (GPBAR-1) is highly expressed in intestine, brown adipose tissue, macrophages/monocytes, spleen and gallbladder [33]. It regulates biliary homeostasis and related metabolism, prevents gastrointestinal and liver inflammation, and is considered a candidate target for improving obesity, dyslipidemia, type 2 diabetes and nonalcoholic fatty liver disease [34,35,36]. Pentacyclic triterpenoids such as betulinic acid and oleanolic acid exhibit TGR5 agonist capacity, possibly due to their structural similarity to bile acids [37,38]. Glycyrrhizic acid, a triterpenoid saponin, can increase GLP-1 secretion via activating TGR5 in intestinal NCI-H716 cells and type 1-like diabetic rats [13]. In this work, TSE1 was found to increase the TGR5 protein expression by approximately 30–80% in a dose-dependent manner (Figure 4). Silence of TGR5 obviously counteracted the elevated GIP mRNA expression in the STC-1 cells induced by TSE1 (Figure 5). Our data revealed for the first time that TGR5 was involved in the GIP regulation, which might be a common mechanism of oleanane-type saponins to stimulate GIP secretion.

Network pharmacology is an effective strategy for identifying drug components and their global mechanisms [39]. In the present study, the STRING and GeneMANIA databases were used to analyze the protein-protein relationship, and six key proteins, ISL1, GIPR, GPR119, GCG, FFAR1 and PCSK1, were found to be associated with SGLT1, TGR5 and GIP. KEGG enrichment analysis showed that these proteins were mainly involved in insulin secretion, cAMP signaling and neuroprotection (Figure 6). ISL1, a DNA-binding transcriptional activator, not only regulates the gene expression of insulin and glucagon to maintain the glucose homeostasis, but also plays a key role in cell proliferation, differentiation and tumorigenesis [40]. GIPR activation promotes insulin secretion through increasing cAMP and proinsulin gene transcription, and regulates pancreatic beta cell survival and proliferation independent of insulin [41]. GPR119 and FFAR1 are free fatty acid sensors that mediate the release of incretin hormones and insulin [42,43]. Glucagon from α-cells was found to be stimulated by GIP in hyperglycemia of Type 2 diabetics, but not in healthy people [44]. PCSK1, mainly expressed in neuronal and endocrine cells, could cleave many protein precursors involved in energy homeostasis [45]. Downregulation of PCSK1 suppresses the generation of GIP and GLP-1 in STC-1 cells [46]. These proteins are involved in tea seed saponin-induced GIP secretion and related physiological functions, and should be further studied as the targets of these saponins in the future.

## 5. Conclusions

In summary, total tea seed saponins and individual saponin compound TSE1 could stimulate GIP mRNA and protein expression in normal mice and STC-1 cells. Inhibition of SGLT1 by phlorizin or silence of TGR5 by siRNA both reverse the TSE1-induced upregulation of GIP expression. Network pharmacology analysis indicated that ISL1, GIPR, GPR119, GCG, FFAR1 and PCSK1 were involved in the regulation of SGLT1, TGR5 and GIP by TSE1. These genes participate in insulin secretion, cAMP signaling, neuroprotection, carbohydrate digestion and absorption, and mineral absorption, suggesting that tea seed saponins possess these bioactivities and are worthy of further study as functional food ingredients or drugs for improving metabolic diseases.

## Data Availability

Not applicable.

## Supplementary Materials

The following supporting information can be downloaded at: https://www.mdpi.com/article/10.3390/nu14163413/s1, Table S1: Five significantly enriched KEGG pathways and involved genes.
